# Effects of time-restricted exercise on activity rhythms and exercise-induced adaptations in the heart

**DOI:** 10.1038/s41598-023-50113-4

**Published:** 2024-01-02

**Authors:** Michael B. Dial, Elias M. Malek, Greco A. Neblina, Austin R. Cooper, Nikoleta I. Vaslieva, Rebecca Frommer, Magdy Girgis, Buddhadeb Dawn, Graham R. McGinnis

**Affiliations:** 1 Department of Kinesiology and Nutrition Sciences, University of Nevada, Las Vegas, 4505 S. Maryland Parkway, Bigelow Health Sciences (BHS) Building 323, Las Vegas, NV 89154 USA; 2 Department of Internal Medicine, Kirk Kerkorian School of Medicine, University of Nevada, Las Vegas, Las Vegas, NV USA

**Keywords:** Cardiac hypertrophy, Circadian rhythms, Cardiac hypertrophy

## Abstract

Circadian rhythms play a crucial role in the regulation of various physiological processes, including cardiovascular function and metabolism. Exercise provokes numerous beneficial adaptations in heart, including physiological hypertrophy, and serves to shift circadian rhythms. This study investigated the impact of time-restricted exercise training on exercise-induced adaptations in the heart and locomotor activity rhythms. Male mice (n = 45) were allocated to perform voluntary, time-restricted exercise in the early active phase (EAP), late active phase (LAP), or remain sedentary (SED) for 6 weeks. Subsequently, mice were allowed 24-h ad libitum access to the running wheel to assess diurnal rhythms in locomotor activity. Heart weight and cross-sectional area were measured at sacrifice, and cardiac protein and gene expression levels were assessed for markers of mitochondrial abundance and circadian clock gene expression. Mice rapidly adapted to wheel running, with EAP mice exhibiting a significantly greater running distance compared to LAP mice. Time-restricted exercise induced a shift in voluntary wheel activity during the 24-h free access period, with the acrophase in activity being significantly earlier in EAP mice compared to LAP mice. Gene expression analysis revealed a higher expression of *Per1* in LAP mice. EAP exercise elicited greater cardiac hypertrophy compared to LAP exercise. These findings suggest that the timing of exercise affects myocardial adaptations, with exercise in the early active phase inducing hypertrophy in the heart. Understanding the time-of-day dependent response to exercise in the heart may have implications for optimizing exercise interventions for cardiovascular health.

## Introduction

Circadian rhythms, derived from the Latin terms ‘circa’ and ‘diem’, describe the ~ 24-h oscillations in various physiological processes, synchronized by the cycles of light and dark. These rhythms confer a selective biological advantage, allowing organisms to synchronize or partition various cellular processes to discrete periods of time at which they are most efficient^1^. Circadian rhythms are driven by the molecular time-keeping mechanism known as the circadian clock, which is present in almost every cell type that has been tested^2,3^. The circadian clock is driven in the positive arm by the transcription factors brain and muscle arnt-like 1 (*Bmal1*) and circadian locomotor output cycles kaput (*Clock*), which initiate the expression of their own negative regulators, Period (*Per1/2*), Cryptochrome (*Cry1/2/3*), and *Rev-erbα*^4^. While this molecular time-keeping mechanism resides in most tissues, its role in the heart is critical to the regulation of cardiovascular function and metabolism^5^.

Previous studies have shown robust circadian rhythms in the responsiveness to various physiological and pathophysiological stressors in the heart. Perhaps most widely appreciated is the temporal pattern in myocardial injury including ischemia–reperfusion injury and cardiac arrest, which peaks at the transition from the rest phase to the active phase (early light phase in diurnal humans, early dark phase in nocturnal rodents)^6–9^. This circadian regulation was recently highlighted in a position statement from the European Society of Cardiology^10^. Durgan et al. revealed that the circadian pattern in ischemia–reperfusion injury severity was dependent on the cardiomyocyte circadian clock in mice, and the rhythm was abolished when the clock was disrupted^6^. Subsequent studies have provided evidence for a strong circadian influence on transcriptional^11,12^ and metabolic control of the heart^13–15^. Cardiac growth or hypertrophy can occur in both physiological and pathological contexts, in response to exercise or hypertension, respectively, for example. Exercise-induced cardiac hypertrophy is considered a beneficial adaptation produced by endurance training, leading to increased heart size and function^16^. Interestingly, the heart appears to be sensitive to pathological hypertrophic stimuli in a time-of-day dependent manner^13,17^. Treating mice with isoproterenol sufficiently induced hypertrophy when administered at zeitgeber time (ZT) 0, corresponding to the active-to-rest transition, but not at ZT12 (rest-to-active transition). Similarly, acute branched chain amino acid feeding also promotes cardiac growth when fed at the end of the active period (ZT20–ZT24), but not in the earlier part of the day^18^. In both cases, cardiac growth was associated with markers of pathological hypertrophy (e.g.—atrial naturetic peptide (*Anf*) expression). However, the temporal gating of physiological hypertrophy, in response to exercise for example, has yet to be thoroughly investigated.

**Figure 1:**
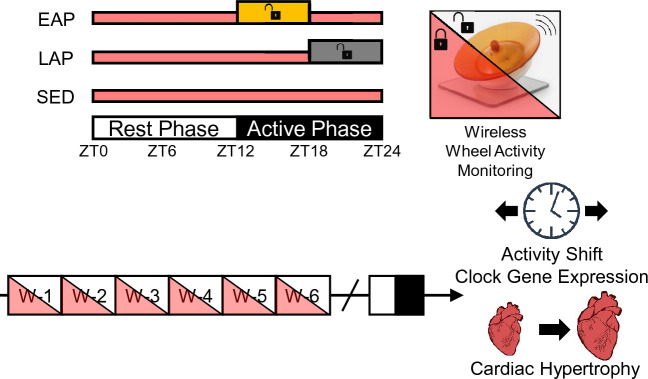
Protocol for time-restricted exercise (TRE) implementation. Mice were housed with wireless running wheels available during the early active period (EAP) or late active period (LAP), or remained sedentary (SED), for a 6-week training period (n = 10 mice/group). At the conclusion of the training period, mice were given ad *libitum* access to the wheel for a 24-h period to determine the effects of time-restricted exercise training on activity rhythms. Hearts were excised to examine the effects of TRE on circadian clock genes and cardiac hypertrophy.

Only recently has the temporal response to exercise been pursued at the molecular level, and most predominantly in skeletal muscle. Several recent studies have determined strong time-of-day dependent regulation of acute exercise-induced transcriptional, metabolic, and proteomic responses^19–22^. However, comparably little is known about the time-of-day dependent response to exercise in the heart. Schroeder et al. determined that voluntary wheel running restricted to the late active phase (ZT18–ZT24) was sufficient to delay the circadian rhythm in heart rate^23^. Additionally, Seo et al., using forced treadmill exercise in rats, showed rather modest time-of-day dependent regulation of the exercise-responsive cardiac proteome^24^. However, in both cases, other exercise-induced adaptations including cardiac hypertrophy were either not evaluated^23^, or not significant^24^. The latter raises the question of exercise dose, where rats were only required to perform a relatively small amount of treadmill exercise (60 min/day) at a moderate relative intensity (20 m/min). Previous studies have shown that mice performing voluntary wheel running exercise will achieve much greater doses of exercise^25,26^, potentially leading to more robust cardiac adaptations. As such, the purpose of this study was to determine the impact of 6-weeks of voluntary wheel running exercise restricted to the early or late active periods on exercise-induced adaptations in the heart (Fig. 1)

## Results

### Time-restricted exercise causes disparate voluntary training volumes between early and late active period

**Figure 2:**
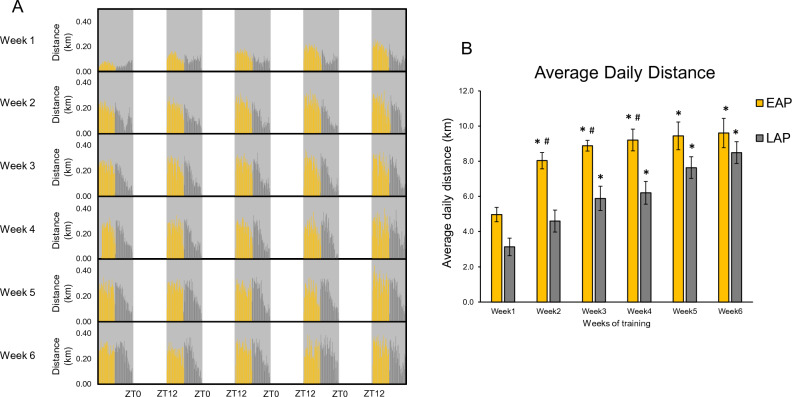
Activity patterns and wheel running volume during 6 weeks of TRE. Actogram representing the 6-week training period (n = 9–10 mice/group; **A**). Wheel data was summed in 10-min bins for EAP (yellow) and LAP (gray) mice. Shaded areas represent the dark cycle (ZT12-ZT24), wheels were locked during the light cycle (ZT0–ZT12). Average daily running distance was calculated as an average daily distance for each week of the 6-week training period for EAP and LAP mice (n = 9–10 mice/group; **B**). Distance represents only wheel activity during the 6-h period of wheel access. ^#^Different from LAP, p < 0.05; *different from Week 1, p < 0.05.

Mice performed 6 weeks of voluntary wheel running, for 5 days per week, which was successfully isolated to either ZT12–ZT18 (EAP) or ZT18–ZT24 (LAP) via manual wheel locking. An actogram of the average wheel running distance for EAP and LAP mice over the 6 weeks of exercise is shown in Fig. 2A. Though no significant group × time interaction was observed, analysis of wheel activity revealed a significant effect of time (p < 0.0001), indicating that both groups increased distance over the course of the study. There was also a significant group effect (p = 0.0021), indicating that EAP mice ran greater distance than LAP mice. Subsequent unpaired, two tailed t-tests revealed differences in average daily distance between EAP and LAP for week 2 (EAP: 8.03 ± 0.47 km vs LAP: 4.68 ± 0.70 km, p < 0.01), week 3 (EAP: 8.88 ± 0.30 km vs LAP: 5.98 ± 0.76 km, p = 0.01), and week 4 EAP: 9.21 ± 0.62 km vs LAP: 6.40 ± 0.69 km, p = 0.04), indicating that EAP ran longer distance than LAP for the beginning of the intervention (Fig. 2B).

### Time-restricted exercise induces a shift in voluntary wheel activity during 24-h free access period

**Figure 3:**
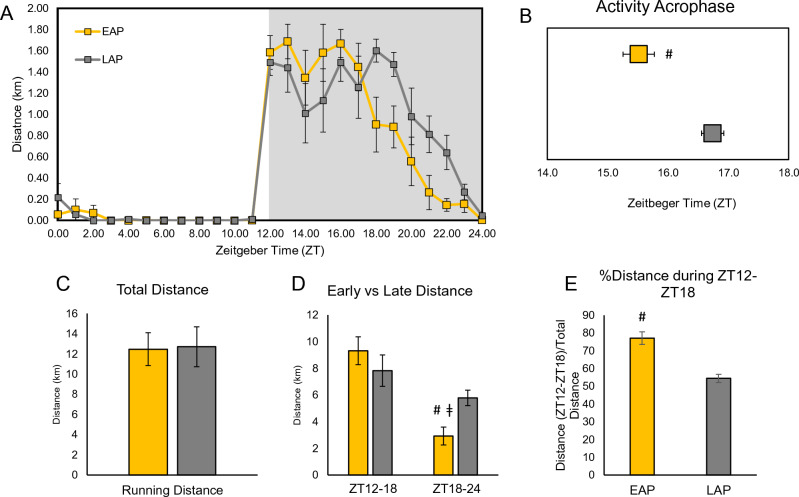
Time-restricted exercise leads to a shift in activity acrophase between EAP and LAP mice. Actogram of a single 24-h period of free access to wheels after 6 weeks of TRE where activity was averaged into 1-h bins (n = 9–10 mice/group; **A**). Acrophase of activity in EAP and LAP mice determined via Cosinor analysis of activity data recorded in 10-min bins (n = 9–10; **B**). Total wheel running distance of EAP and LAP mice during the 24-h period of free wheel access (n = 9–10 mice/group; **C**). Distance run during the first 6 h of the active phase (ZT12–18) and the last 6 h of the active phase (ZT18–24) during ad libitum wheel access of EAP and LAP mice (n = 9–10 mice/group; **D**). Percent of total activity completed in the first 6 h of the active phase of EAP and LAP mice (n = 9–10 mice/group; **E**). ^#^Different from LAP, p < 0.05; ^‡^different from ZT12–ZT18, p < 0.05.

To evaluate the effects of time-restricted exercise on locomotor activity rhythms, EAP and LAP were allowed a single 24-h period (12 h Light, 12 h Dark) of unrestricted access to the running wheel after 6 weeks of training. An actogram of the average wheel running distance of EAP and LAP mice (presented with 1-h bin) is shown in Fig. 3A. Cosinor analysis of wheel activity data revealed significant differences in the acrophase of EAP mice vs LAP mice (EAP: 15.22 ± 0.25 vs LAP: 16.74 ± 0.19, p < 0.001; Fig. 3B). While activity timing was discrepant between groups, we found that there was no significant difference in the total volume of activity during the 24-h free access period (EAP: 12.46 ± 1.63 km vs LAP: 12.72 ± 1.98 km, p = 0.92, Fig. 3C). We subsequently determined what absolute, and what proportion of distance was performed during the early and late portions of the active phase (Fig. 3D,E, respectively). EAP mice performed the majority of their activity during the first 6 h of the active period (9.31 ± 1.05 km vs 2.91 ± 0.67 km, p < 0.01), while LAP activity was more evenly distributed between the entire active period (7.81 ± 1.17 km vs 5.78 ± 0.58, p < 0.05). Finally, the proportion of total activity performed during the first 6 h of the active phase was significantly higher in EAP mice compared to LAP mice (78.35 ± 2.96% vs 56.10 ± 2.16%, p < 0.01).

### Time-restricted exercise exerts modest effect on myocardial circadian clock gene expression

**Figure 4:**
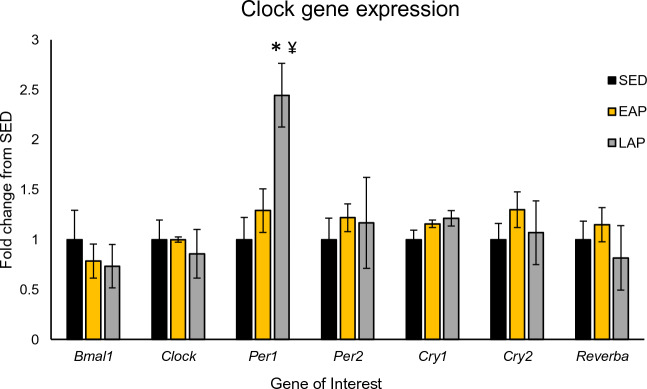
Clock gene expression in the heart was modestly impacted by time-restricted exercise. Expression of myocardial *Bmal1*, *Clock*, *Per1*, *Per2*, *Reverb-*α, *Cry1*, and *Cry2* in RNA isolated from hearts of mice sacrificed at ~ ZT13.5, after 6 weeks of TRE (n = 4–5 mice/group). *Different from SED, p < 0.01; ^¥^different from EAP, p < 0.05.

We subsequently measured the expression of canonical circadian clock genes in the hearts of mice after the 6-week time-restricted exercise intervention (n = 5 mice/group, Fig. 4). All mice were sacrificed at ~ ZT13.5, to compare the effects of exercise on circadian clock gene expression levels independent of time of day. Following a one-way ANOVA (p = 0.0053), post-hoc analysis revealed a significantly higher mRNA expression of *Per1* in LAP mice compared to SED (LAP: 2.45 ± 0.64 vs SED: 1.00 ± 0.50, p < 0.01) and EAP (LAP: 2.45 ± 0.64 vs EAP: 1.29 ± 0.49, p = 0.02), suggesting that later exercise has a greater impact on myocardial circadian clock gene expression. No other effects or interactions were observed for other circadian clock genes.

### Early active phase exercise elicits greater cardiac hypertrophy compared to late active phase

**Figure 5:**
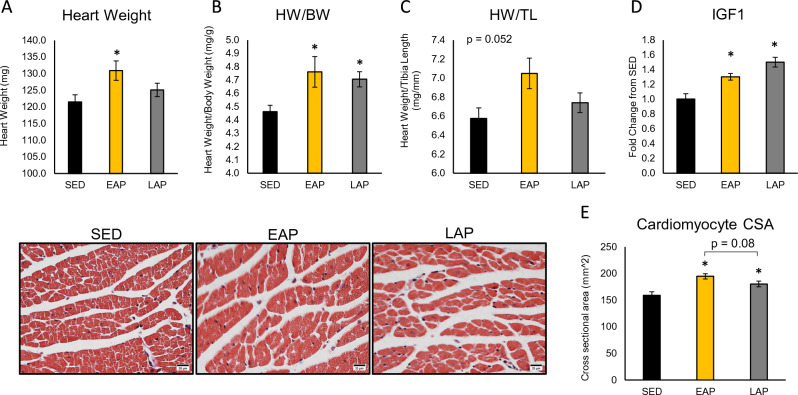
EAP exercise preferentially elicits cardiac hypertrophy. Absolute heart weight (**A**), heart weight standardized to body weight (**B**), heart weight standardized to tibia length (**C**) was determined in hearts isolated from mice after 6 weeks of TRE (n = 8–9 mice/group). Gene expression for insulin-like growth factor 1 *(IGF-1*) was elevated in both exercise groups (n = 4–5 mice/group; **D**). Cardiomyocyte cross-sectional area was increased in EAP and LAP hearts compared to SED (n = 5 mice/group; **E**). Scale bar is 20 µm. *Different from SED, p < 0.05.

Frozen hearts were weighed immediately before processing to evaluate gravimetric changes following exercise (n = 8–10 mice/group). Heart weights are presented as raw tissue weight (Fig. 5A), as well as normalized to body weight (HW/BW; Fig. 5B) and tibia length (Fig. 5C). One-way ANOVA analysis revealed a significant effect of exercise on raw heart weight (p = 0.041). Post-hoc comparisons revealed significantly higher heart weight in EAP mice compared to SED (EAP: 130.90 ± 3.2 mg vs SED: 121.4 ± 3.4 mg, p < 0.05), with no significant differences in LAP mice. Similar results were observed when heart weight was normalized to body weight (p = 0.044), with post-hoc comparisons revealing higher HW/BW in EAP mice (EAP: 4.8 ± 0.1) and LAP mice (4.7 ± 0.1) compared to SED (4.5 ± 0.1, both p < 0.05). When normalized to tibia length, the ANOVA approached, but did not reach statistical significance (p = 0.052). Histological assessment of cardiac hypertrophy was performed on a separate cohort of mice (n = 5 mice/group). Cardiomyocyte cross sectional area (CSA) was increased in EAP mice and LAP mice compared to SED mice (SED = 158.9 ± 6.6, EAP = 194.6 ± 5.0, LAP = 180.0 ± 5.4; p < 0.05; Fig. 5E). While CSA was numerically higher in EAP mice compared to LAP, the effect was not statistically significant (p = 0.08). Expression of a molecular marker of hypertrophy, *IGF1* (Fig. 5D), was increased at the mRNA level in EAP and LAP hearts compared to SED (SED: 1.00 ± 0.07 vs EAP: 1.30 ± 0.045 vs LAP: 1.50 ± 0.07, p < 0.05).

### Time-restricted exercise modestly impacts myocardial mitochondrial abundance

**Figure 6:**
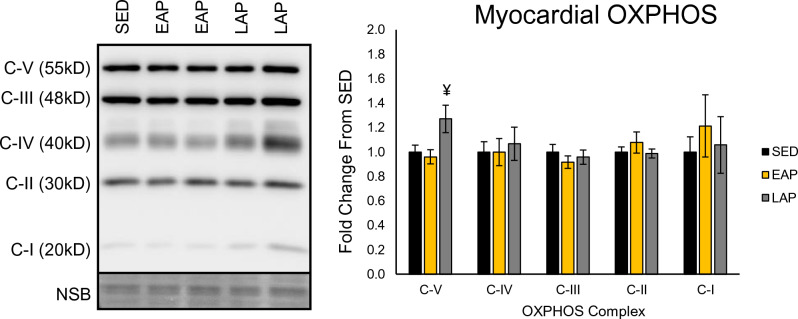
Oxidative phosphorylation complex protein expression is modestly impacted by voluntary wheel running. Western blot quantification OXPHOS complexes I–V and representative western blot image (n = 9–10 mice/group). ^¥^Different from EAP, p < 0.05.

To measure potential time-of-day dependent changes in exercise-induced mitochondrial abundance, cardiac tissue was evaluated for expression of component proteins found in the each of the five oxidative phosphorylation (OXPHOS) complexes (n = 8–10 mice/group, Fig. 6). No significant differences between groups were observed for Complexes I–IV. However, we observed a significant effect of exercise via one-way ANOVA on Complex V (p = 0.0216). Post-hoc comparisons revealed that exercise in the late active phase resulted in a significantly increased expression of Complex-V (ATP synthase subunit alpha), compared to EAP (EAP: 0.96 ± 0.06; LAP: 1.27 ± 0.11, p = 0.03), while EAP exercise did not (SED: 1.00 ± 0.06 vs EAP: 0.96 ± 0.06, p = 0.94). SED was not significantly different compared to LAP (SED: 1.00 ± 0.06 vs LAP: 1.27 ± 0.11, p = 0.0668). Taken together, these data indicate that mitochondrial adaptation in the heart was modestly affected by the timing of exercise, which a potentially greater response when exercise was performed in the late active period.

## Discussion

The purpose of this study was to evaluate the effect of the timing of exercise (EAP vs LAP) on cardiac hypertrophy and circadian regulation. One major finding of the present study was that myocardial hypertrophy only occurred when exercise was performed in the early active period and not in the late active period. Chronic endurance exercise results in physiologic, eccentric hypertrophy of the heart, characterized by a concomitant increase in the left ventricular wall thickness, as well as left ventricular volume^27^. In both mice and humans, at least 3–4 weeks of training is necessary to cause a 5–20% increase in heart weight^28–31^. Exercise-induced myocardial hypertrophy is usually limited to a 10–15% increase in heart mass in humans^27,32^. In rodents, physiologic myocardial hypertrophy ranges from 5 to 24% for absolute heart weight^33–36^. In the present study, EAP exercise resulted in a 7.8% increase in absolute heart weight, while LAP exercise did not cause significant hypertrophy. Furthermore, there was a trend for cardiomyocyte CSA to be higher in EAP mice, though it was significantly increased in both exercise groups. While this is the first study to identify time-of-day dependent regulation of physiological cardiac hypertrophy resulting from exercise, interventions inducing pathological hypertrophy seem to be more effective during the late portion of the active period^14,18^. Given the different nature of the cardiovascular adaptations, it is possible that an alternative circadian time period is more sensitive to physiological cardiac hypertrophic stimuli. It is also possible that a dose-dependent increase in cardiac hypertrophy was seen, where the larger volume of training done by EAP mice elicited greater hypertrophy. While this is possible, other studies have shown significant cardiac hypertrophy in a period of 2 weeks^37^. During the weeks 5 and 6 of our study, here was not a significantly different distance ran by EAP and LAP mice, suggesting that an equal exercise stimulus occurred for the final 2 weeks. Future studies using distance clamped voluntary exercise, as well as functional assessment of cardiac health (i.e.—echocardiography) should be performed.

Another finding of the present study was that, particularly in the first portion of the study, mice performed less running during the late active period compared to the early active period. This is in contrast to Schroeder et al., who found that exercise consolidated to these times of day resulted in similar volumes between groups^23^. Other studies investigating time-restricted exercise found that after a 2-week habituation period of free access to a running wheel, both early and late exercise groups ran similar distances^38^. As the EAP and LAP mice in the current study approached similar training volume by the end of the 6-week period, it is likely that a run-in habituation period, similar to Adamovich et al., would have further limited those differences. Our results also agree with a time-restricted exercise study done in rats^39^, in which rats were restricted to exercising for 90 min during the early or middle active period. Initially, early exercisers ran substantially less than the middle active period rats. After 6 days, the groups were not different^39^. Boakes et al. also observed that rats given a fixed daily exercise time ran greater distances than those placed on a variable schedule^39^. Taken together, these collective findings suggest that there is a learning effect of restricting exercise to different times of day.

The 24-h free access period yielded findings pertinent to whole-body activity rhythms in the present study. We observed that the acrophase of activity was significantly later in LAP mice compared to EAP mice, indicating that activity rhythms were shifted by their habitual exercise time to some extent. Previous studies have shown that timed exercise (via forced treadmill running or limited wheel access) can shift activity rhythms^40^. Wolff and Esser demonstrated altered activity patterns in mice when exercised during the light phase^41^. Maier et al. were also able to significantly advance activity rhythms of mice on a skeleton photoperiod by giving them access to a running wheel 12 h earlier in the day^20^. Alternatively, Adamovich et al., using early (ZT12–ZT18) and late exercise (ZT18–ZT24), failed to produce a significant difference in activity rhythms between the exercise groups, though they were both different from a control sedentary group^38^. Schroeder, et al. showed a much larger shift in activity using the same scheduled wheel availability (~ 5 h difference in acrophase between early and late active phase exercise)^23^. However, their activity rhythms were recorded over 10 days, during the wheel lock schedule, compared to our ad libitum wheel access design which only revealed an ~ 1.5 h difference. It is possible that a longer duration of time-restricted exercise, or the implementation of forced rather than voluntary exercise, may further differentiate activity acrophase, which could be used to supplement circadian alignment.

The present study demonstrated that LAP exercise caused an increase in *Per1* expression in the heart, suggesting that the timing of exercise influences the intrinsic cardiomyocyte circadian clock, albeit to a minor extent. It is also interesting that this effect was noted only in *Per1*, and not other clock genes. One possible explanation is that cardiac *Per1* expression is dependent on adrenergic signaling^42^. In fact, treatment of hearts with the β-adrenergic activator isoproterenol, which mimics the increased contractility and work that occurs with exercise, increased *Per1* expression without impacting other clock genes^42^. A major limitation of this finding is that mice were sacrificed only at one time of day (~ ZT12.5–14.5), and not throughout a time-course of tissue collection. However, previous studies have used time-of-day independent changes in clock gene expression as a reference for the effect of exercise on the circadian clock in peripheral tissues. Bruns et al. showed that free-access to a running wheel running was capable of altering *Per2* and Clock gene expression in the left ventricle of old female mice, but no effects were seen in younger male mice, similar to the age used in this study^43^. Exercise has been shown to modulate expression of clock components in human skeletal muscle, for example, where upregulation of *Per1* and *Per2* occurred after ~ 30–45 min intense, isotonic exercise in the morning^44^. Similar increases in *Per2* expression in skeletal muscle have also been observed after both acute aerobic and resistance exercise^45^. Using the *Per2* luciferase reporter mouse, studies have also shown the phase shifting ability of exercise in peripheral tissues^40,46,47^, however, this technique has not been used in the heart. More studies into how exercise affects the cardiomyocyte circadian clock are needed to better understand its influence.

These data indicate that exercise-induced adaptations in the heart vary based on the time-of-day exercise is performed. Exercising in the ‘morning’ (early active period) preferentially favored cardiac hypertrophy compared to ‘evening’ exercise (in the later active period). Exercise in the latter portion of the active period has a greater impact on circadian clock gene expression in the heart, and diurnal locomotor activity rhythms. Targeting exercise prescription to different times of day may be used to preferentially elicit different adaptations and potentially entrain the circadian rhythm. These data may hold important implications for clinical applications like cardiac rehabilitation, where maximizing exercise-induced improvements in cardiovascular function could be used to improve patient outcomes, which requires future study.

## Methods

### Mice

The present study used male C57Bl/6J mice (n = 45, starting at 12 weeks of age, Jackson Laboratories). Mice were housed on a tightly controlled 12–12 light–dark cycle (ZT0/Lights ON at 8:00 pm, ZT12/Lights OFF at 8:00 am) in temperature and humidity regulated rooms with ad libitum access to food and water throughout the experimentation period. All animal handling during the study period occurred during the active period of nocturnal rodents (i.e.—ZT12–ZT24, during the dark phase) under dim red light to avoid circadian influences of light, and to more appropriately synchronize exercise training/testing with the active cycle of nocturnal rodents. All methods were carried out in accordance with relevant guidelines, approved by the Institutional Animal Care and Use Committee at the University of Nevada Las Vegas (IACUC-01211), and comply with ARRIVE Guidelines.

### Time-restricted exercise

Mice were allocated to three experimental groups (n = 15 mice/group); Sedentary (SED, locked wheel to control for cage enrichment), Early Active Phase Exercise (EAP, access to a running wheel for the first 6 h of the active period from ZT12–ZT18), or Late Active Phase Exercise (LAP, access to a running wheel for the last 6 h of the active period from ZT18-ZT24), 5 days/week for 6 weeks (Fig. 1). One SED mouse developed malocclusion and was excluded from the study, so the resulting sample size for SED was n = 14. Wheels were manually locked to prevent activity outside of the prescribed window. Wheel activity was recorded using wireless enabled running discs (Med Associates, Fairfax, VT) to confirm temporal isolation of running wheel exercise, and to evaluate the volume of exercise performed. Wheel revolutions were recorded using Wheel Analysis Software (Med Associates, Fairfax, VT). To evaluate temporal distribution of wheel activity, wheel revolutions were converted to distance and summed in 10-min bins. Additionally, average distance/activity period was shown throughout each week of the study period (Fig. 2).

### Ad libitum 24-h activity

After 6 weeks of the Time-Restricted Exercise schedule, mice were allowed a single day of 24-h ad libitum access to an unlocked running disc (n = 9–10 mice/group). Wheel activity was recorded in 10-min bins throughout the 24-h period in 12:12 LD conditions. Wheel activity was presented in 1-h bins (Fig. 3A). Cosinor analysis was performed on data recorded every 10 min to determine activity acrophase for each mouse using Cosinor Online^48^, and compared between EAP and LAP mice to evaluate shifts in activity rhythms (Fig. 3B).

### Euthanasia

At the conclusion of the study, mice were euthanized under isoflurane anesthesia (3% Isoflurane vaporized in 100% O_2_, to effect), at approximately ZT13.5 (ZT12.5–14.5). Food was removed at ZT12 on the day of euthanasia. A surgical plane of anesthesia was confirmed by loss of paw pinch and corneal reflexes, at which time hearts were excised, allowed to beat clear of blood in ice cold saline (0.7% Saline), and snap frozen in liquid nitrogen. Frozen hearts were weighed to assess cardiac hypertrophy elicited by exercise. The order of euthanasia was staggered through experimental groups (i.e.—SED, EAP, LAP, SED, etc.) to minimize confounding differences in the time of day of euthanasia. The average ZT of euthanasia was nearly identical across groups (SED = ZT13.47 ± 0.23, EAP = ZT13.48 ± 0.22, LAP = ZT13.51 ± 0.25).

### Histological assessment of cardiomyocyte cross-sectional area

At the end of the study protocol, mice were euthanized, the heart was excised and perfused retrogradely with 10% neutral-buffered formalin. The left ventricle was sectioned perpendicular to its longitudinal axis, processed, embedded in paraffin and sectioned. Multiple digital images were acquired from Masson’s trichrome-stained myocardial sections using an Olympus BX51 microscope equipped with Olympus DP80 digital camera. Using the NIH ImageJ (version 1.54f) software, cardiomyocyte cross-sectional area was measured in transversely sectioned myocytes with a circular profile and a central nucleus^49^. On average, a total of 80–100 myocytes were measured in each heart.

### Protein isolation and western blotting

Frozen hearts were powdered with a mortar and pestle while cooled in liquid nitrogen. Approximately 20 mg of powdered heart tissue was mixed with lysis buffer with protease and phosphatase inhibitors, then vigorously vortexed for 10 s at maximum speed, and sonicated for 10 s. After a 30-min incubation, homogenates were centrifuged at 12,000×*g* for 10 min at 4 °C, and lysate supernatants were aliquoted for protein concentration determination using the Pierce BCA assay (Thermo Fisher Scientific, # 23225). Proteins were normalized to 1 µg/µL in Laemmli buffer with 2-Mercaptoethanol. Non-denatured western blot samples were used for the determination of oxidative phosphorylation (OXPHOS) complex abundance using a primary antibody cocktail (Abcam, ab110413). Proteins were separated on 10% acrylamide gels and transferred onto methanol activated PVDF membranes overnight at 4 °C. Successful transfer and even protein loading were confirmed with Memcode Reversible Protein Stain (Thermo Scientific #24580), and a non-specific protein band (NSB) for this stain was used for normalization. This use of chemiluminescent normalizing proteins (e.g.—β-actin or Tubulin) is prohibitive since the lysates for OXPHOS western cannot be heat denatured, per manufacturer’s instructions. Membranes were blocked with 5% NFDM in TBST for 1 h before primary antibodies diluted in 5% BSA based blocking were applied overnight at 4 °C. Secondary antibodies diluted in 5% NFDM in TBST were applied for 1 h at room temperature before imaging with HRP-substrate (Luminata Forte) using a Biorad ChemiDoc Imaging system (Bio-Rad, Hercules, CA). Raw unaltered exposures for all western blot images can be found in the Supplementary Information.

### RNA isolation and PCR

RNA was isolated using conventional Trizol techniques. Briefly, ~ 20 mg of powdered heart tissue was homogenized in Trizol using a BeadBeater for 2 min at maximum speed (BioSPEQ, Irvine, CA). RNA concentration and quality was checked using a NanoDrop 2000, and 1000 ng of RNA was used for cDNA library preparation. RT-PCR was performed with 25 ng cDNA in a master mix containing gene specific primers and SYBR Green (PerfeCta # 95072-250), tracked through 40 PCR cycles. Canonical circadian clock genes were evaluated using the following primer sequences: *Bmal1* Fʹ—CACCAACCCATACACAGAAG*, Bmal1* Rʹ—GGTCACATCCTACGACAAAC, *Per1* Fʹ—CAGACCAGGTGTCGTGATTAAA, *Per1* Rʹ—CGAAACAGGGAAGGTGAAGAA, *Per2* Fʹ—ATGAGTCTGGAGGACAGAAG, *Per2* Rʹ—CCTGAGCTGTCCCTTTCTA, *Clock* Fʹ—ACTCAGGACAGACAGATAAGA, *Clock* Rʹ—TCACCACCTGACCCATAA, *Rev-erb-α* Fʹ—TGGCCTCAGGCTTCCACTATG, *Rev-erb-α* Rʹ—CCGTTGCTTCTCTCTCTTGGG, *Cry1* Fʹ—AGAGGGCTAGGTCTTCTCGC, *Cry1* Rʹ—CTACAGCTCGGGACGTTCTC, *Cry2* Fʹ—GTCTGTGGGCATCAACCGA, *Cry2* Rʹ—TGCATCCCGTTCTTTCCCAA*.* Expression of *IGF1* was evaluated as a molecular marker for cardiac hypertrophy using the following primer sequences: IGF1 Fʹ—TACTTCAACAAGCCCACAGG, *IGF1* Rʹ—TCTCCAGTCTCCTCAGATCAC. Gene expression was quantified using the 2^∆∆Ct^ method and reported as ‘fold change from SED’ for each gene of interest.

### Statistical analysis

To assess the differences in average daily distance in wheel activity for each week between EAP and LAP groups, we performed a two-way repeated-measures ANOVA (group: [EAP, LAP] × week [1, 2, 3, 4, 5, 6]). To analyze differences between groups, multiple unpaired, two-tailed t-tests with Bonferroni correction were performed. Additionally, to determine the effects of time-restricted exercise on voluntary activity, the acrophase of wheel activity was determined during the final day of ad libitum wheel access using Cosinor analysis^48^. The acrophase in wheel activity was compared between EAP and LAP mice using an unpaired, two-tailed t-test. All outcomes for western blotting and PCR are reported as fold change from SED. Values collected via densitometry (for western blotting) or 2^∆∆Ct^ method (for PCR), were each divided by the average of the SED group allowing for the determination of normalized change from the SED group, as well as the appropriate variability around the mean in each group. Values greater than two standard deviations away from the mean were treated as outliers and removed from analysis. Cross-sectional analysis, as well as molecular analyses (protein levels, gene expression), and heart weight between SED, EAP and LAP mice was analyzed using a one-way ANOVA with Tukey’s honestly significant difference post-hoc comparisons. In all cases, significance was accepted at p < 0.05. In the case of post-hoc comparisons (i.e. Bonferroni correction), an adjusted p-value accounting for multiple comparisons was used as the threshold for significance. All data are represented as mean ± standard error of the mean.

### Supplementary Information

Supplementary Information.

## Data Availability

Authors will provide all raw data, as well as access to biological samples, produced within the described experiments upon reasonable request submitted to the corresponding author (GRM).
