# Supplementary figures and images for: Effects of time-restricted exercise on activity rhythms and exercise-induced adaptations in the heart
Source: Sci Rep. 2024 Jan 2;14:146. doi: 10.1038/s41598-023-50113-4 (PMC10761674; doi:10.1038/s41598-023-50113-4)

Gel 1

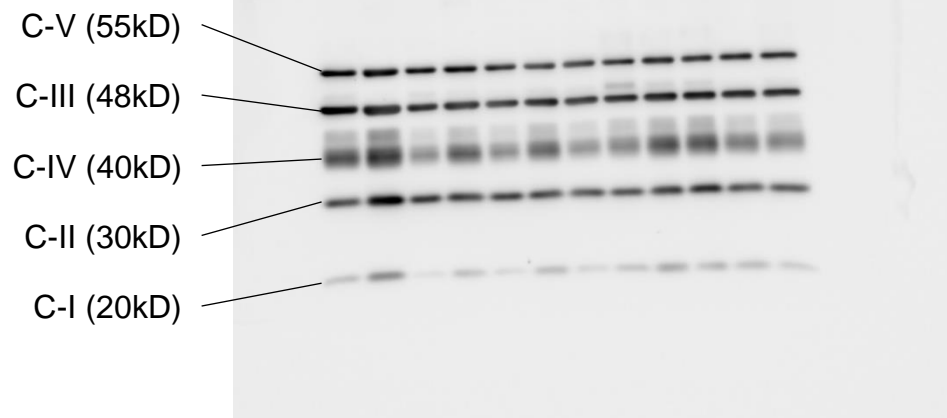

Gel 2

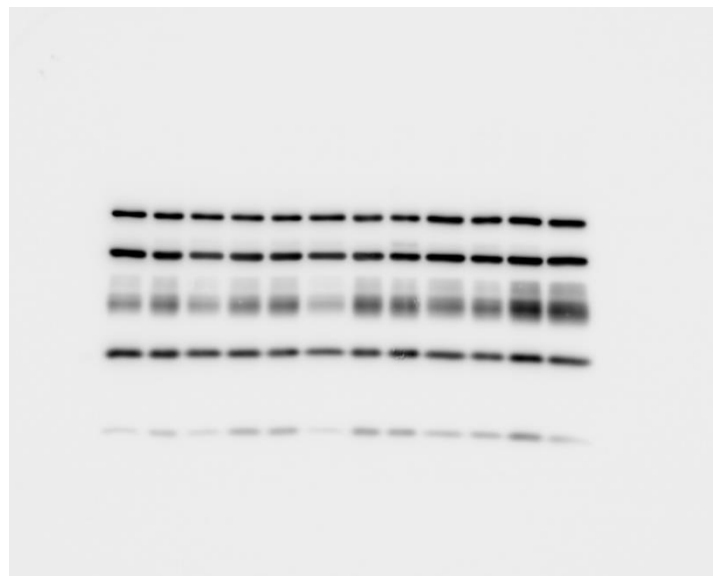

Gel 3 (Representative Image)

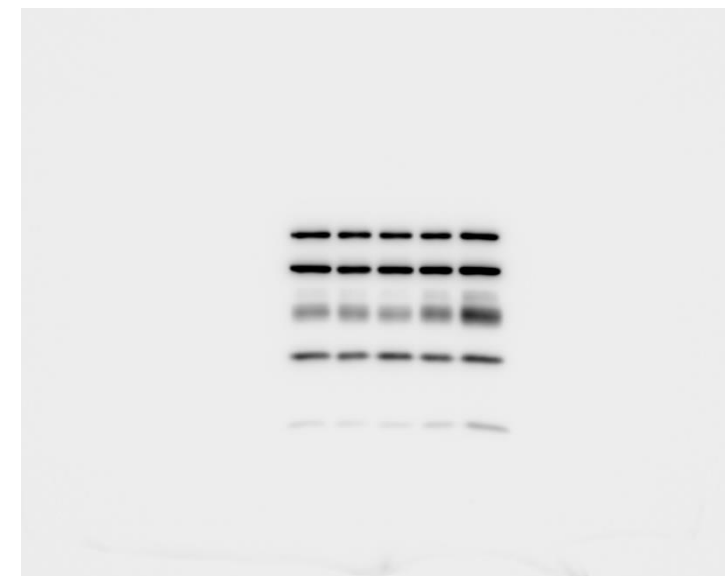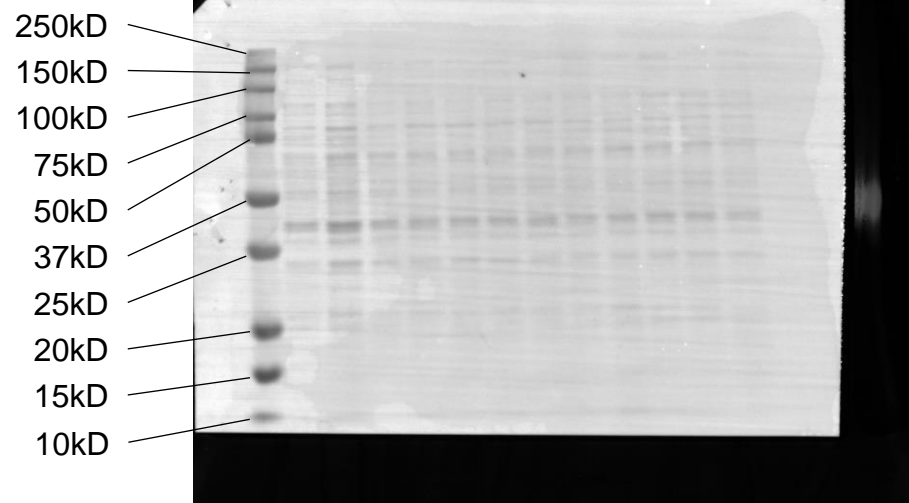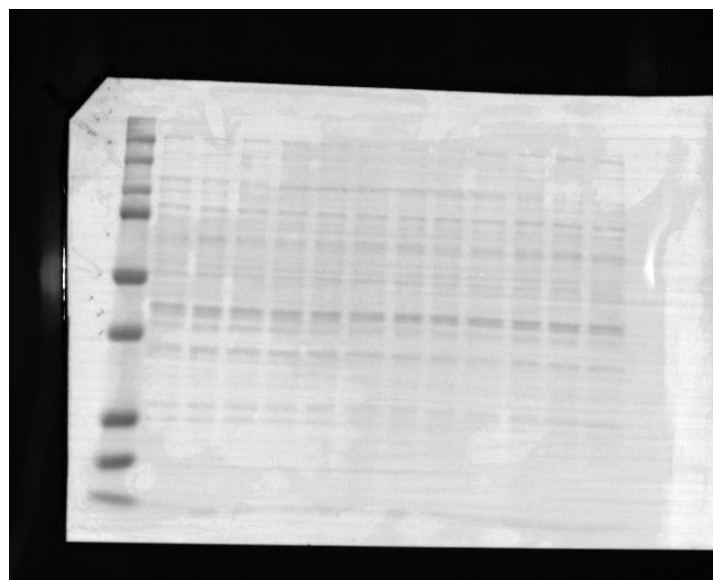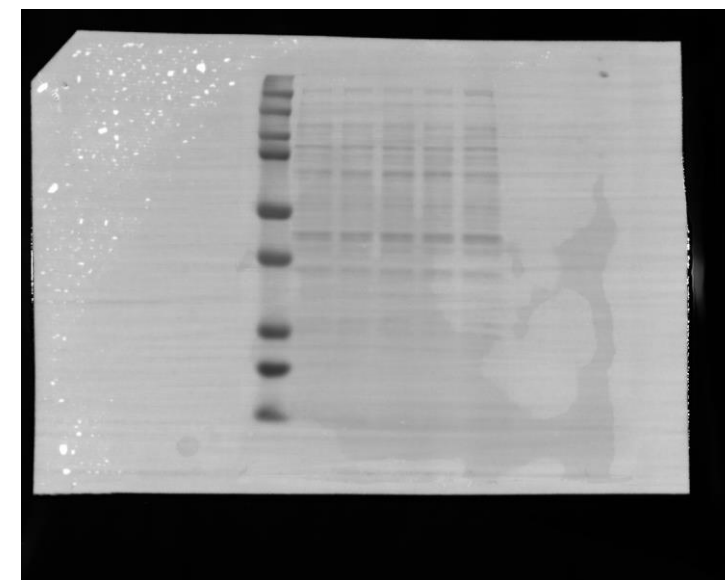

Supplement: Supplementary file 1 — Supplementary Information. [file 41598_2023_50113_MOESM1_ESM.pdf]
